# Exploring the multidimensional impact of caregiver burden in patients with inflammatory bowel disease

**DOI:** 10.3389/fpubh.2025.1528778

**Published:** 2025-05-29

**Authors:** Yuan Yuan, Hao Wang, Xiaomei Song, Wei Tan, Yan Liu, Hongli Liu, Chaoqun Hu, Hong Guo

**Affiliations:** ^1^Chongqing Medical University, Chongqing, China; ^2^Department of Gastroenterology, Chongqing General Hospital, Chongqing University, Chongqing, China

**Keywords:** inflammatory bowel disease, caregiver burden, influencing factors, cross-sectional study, quality of life

## Abstract

**Background:**

Inflammatory bowel disease (IBD) is a chronic, non-specific inflammatory condition characterized by periods of relapse and remission, often requiring frequent medical visits. Family members, who are central to the patient's social support network, often serve as primary caregivers, facing significant physiological, psychological, and financial strain. However, research on caregiver burden in IBD is limited. This study aimed to assess the current state of caregiver burden in IBD and identify contributing factors, providing a valuable reference for evaluating caregiver burden and developing targeted interventions.

**Methods:**

From February to December 2022, we conducted on-site questionnaire surveys with 236 IBD patients and their caregivers. The surveys collected general demographic information and utilized the Self-Rating Anxiety Scale (SAS), Self-Rating Depression Scale (SDS), Pittsburgh Sleep Quality Index (PSQI), and Caregiver Burden Inventory (CBI) to assess key variables. Basic information gathered from IBD patients and their caregivers included age, health status, education level, marital status, monthly family income, the presence of co-caregivers, and daily caregiving duration.

**Results:**

The study included 236 IBD patients and their caregivers. We found positive correlations between CBI scores and scores on the SAS, SDS, and PSQI (*r* = 0.180–0.709, *p* < 0.01). Multiple linear regression analysis revealed that higher caregiver depression and anxiety levels, longer daily caregiving hours, younger patient age, lower patient educational level, and greater disease severity were significantly associated with increased caregiver burden. Female caregivers reported experiencing a greater burden than male caregivers.

**Conclusion:**

Caregivers of IBD patients experience a substantial and multifaceted burden that is frequently underestimated. This excessive burden negatively impacts both patient outcomes and the caregiver's wellbeing, highlighting the critical need for comprehensive support from healthcare professionals and society to effectively address and alleviate caregiver burden.

## 1 Introduction

Inflammatory bowel disease (IBD), encompassing Crohn's disease (CD) and ulcerative colitis (UC), is a chronic, immune-mediated disorder of the gastrointestinal tract characterized by relapsing symptoms; currently, there is no cure (1, 2). Globally, the incidence of IBD has been rising in recent years. Although incidence rates have stabilized in Western countries, several nations in North America, Oceania, and Europe report IBD prevalence exceeding 0.30%, resulting in a considerable impact on societal productivity and patient quality of life (3, 4). China has experienced a notable increase in both the prevalence and incidence of IBD. Projections from the Chinese Center for Disease Control and Prevention estimate that the total number of IBD cases in China will reach 1.50 million by 2025 (5, 6).

IBD is most frequently diagnosed in young adults between 18 and 49 years of age; however, the incidence of pediatric IBD is rising globally (7). A multi-center prospective study conducted by the Canadian Pediatric IBD Network (8) revealed variations in IBD phenotypes across the pediatric age range. The study found that ~19% of children receive an IBD diagnosis before the age of 10, and 4% are diagnosed before the age of 6, indicating a trend toward earlier onset of the disease.

While the life expectancy of patients with UC is comparable to that of the general population, those with CD tend to have a slightly shorter life expectancy, suggesting a prolonged experience with the disease. As a chronic and currently incurable condition, IBD carries a guarded prognosis, characterized by frequent relapses and high rates of hospitalization, which can lead to substantial psychological and practical burdens for both patients and their families (9–11).

Psychological distress is prevalent among IBD patients, requiring caregivers to address not only the patients' physical ailments but also the associated emotional challenges. Consequently, the psychological wellbeing of caregivers warrants careful consideration. Prior research has predominantly concentrated on IBD patients, often neglecting the significant impact of caregivers' physical and mental health on patient care outcomes. Therefore, this study aims to evaluate the psychological status and burden experienced by caregivers of IBD patients, utilizing the Self-Rating Anxiety Scale (SAS), Self-Rating Depression Scale (SDS), Pittsburgh Sleep Quality Index (PSQI), and Caregiver Burden Inventory (CBI). Furthermore, the study will analyze factors that influence caregiver burden, providing insights that may inform strategies to reduce this burden in the context of IBD.

## 2 Materials and methods

### 2.1 Participants

This cross-sectional study employed a convenience sampling method. The caregivers of 236 patients with IBD who received treatment at the Gastroenterology Department of Chongqing General Hospital during February–December 2022 were included in the study. The survey was conducted through on-site distribution and collection of questionnaires, with the consent of the participants. The researchers provided uniform instructions to the participants on how to complete the questionnaires and any precautions to be taken. After the participants completed the questionnaires, the researchers checked for any missing information and completed it on the spot, if necessary.

### 2.2 Inclusion and exclusion criteria

Inclusion criteria: 1) diagnosis of IBD based on the “Consensus on the Diagnosis and Treatment of IBD (Beijing, 2018)” (12); 2) caregivers who were relatives of the patients and had been involved in the care of the patients for a long period (care duration >3 months); 3) caregivers aged 20–80 years; 4) caregivers with basic reading comprehension ability, no history of mental illness or cognitive impairment; 5) informed consent and voluntary participation in the study. Exclusion criteria: 1) occurrence of other major negative events in the family; 2) presence of other major physical or mental illnesses in the family.

### 2.3 Scales and questionnaires

#### 2.3.1 General information questionnaire

The patient's basic information included age, gender, education level, occupation, disease type, disease duration, disease severity, and medical expense payment method. The caregiver's basic information included age, gender, health status, presence of other diseases, education level, occupation, religious beliefs, marital status, relationship with the patient, monthly family income, presence of co-caregivers, and daily caregiving duration.

The severity of IBD was assessed using established clinical-scoring systems for UC and CD. The severity of UC was determined with reference to the Modified Mayo Score (MMS), with a score of < 2 points indicating remission, 3–5 points indicating mild severity, 6–10 points indicating moderate severity, and 11–12 points indicating high severity. The severity of CD was evaluated with reference to the Crohn's Disease Activity Index (CDAI), with a score of < 150 points indicating remission, 150–220 points indicating mild severity, 220–450 points indicating moderate severity, and >450 points indicating high severity (13).

#### 2.3.2 Self-rating anxiety scale (SAS)

SAS was used to assess the severity of anxiety symptoms and their changes during treatment (14). The scale consists of 20 items that primarily assessed the frequency of symptoms defined by each item in the past 2 weeks. Each item was scored on a 4-point scale, and the total score was multiplied by 1.25 to obtain the standard score. The cutoff values for the standard score are as follows: 50–59 for mild anxiety, 60–69 for moderate anxiety, and >69 for severe anxiety. The Chinese version of the SAS has been validated in Chinese populations, demonstrating good internal consistency with a Cronbach's alpha of 0.92 (15).

#### 2.3.3 Self-rating depression scale (SDS)

SDS was used to assess the severity of depression symptoms and their changes during the treatment duration (14). The scale consists of 20 items that primarily assessed the frequency of symptoms defined by each item in the past 2 weeks. Each item was scored on a 4-point scale, and the total score was multiplied by 1.25 to obtain the standard score. According to the Chinese norm of SDS scores, the cutoff values are as follows: 53–62 for mild depression, 63–72 for moderate depression, and >72 for severe depression. The Chinese version of the SDS has demonstrated good reliability and validity with Cronbach's alpha coefficient of 0.83 (16).

#### 2.3.4 Pittsburgh sleep quality index (PSQI)

PSQI was used to assess the sleep quality of patients in the past month (17). The scale consists of 18 items that form seven factors. Each factor was scored from 0 to 3, and the sum of the scores for all factors gives the total score of 0–21. The higher the score, the poorer the sleep quality. The PSQI has been widely used in Chinese sample with satisfied validity and reliability with Cronbach's alpha coefficient of 0.84 (18).

#### 2.3.5 Caregiver burden inventory (CBI)

This scale consists of 24 items (19, 20), including questions on physiological burden (4 items), emotional burden (5 items), social burden (5 items), time-dependency burden (5 items), and developmental burden (5 items), forming five dimensions. A 5-point scale is used, with responses ranging from “strongly disagree” to “strongly agree,” and scores ranging from 0 to 4 for each item. The total score ranges from 0 to 96, with higher scores indicating a heavier caregiving burden. The scores of mild burden, moderate burden, and severe burden were 0–32, 33–64, and 65–96, respectively. The Chinese-version CBI was validated and suggested a satisfactory reliability with Cronbach's alpha coefficient of 0.85 (21).

### 2.4 Sample size estimation

Based on Kendall's method, the sample size for multivariate analysis should be 5 to 10 times the number of variables (22). In this study, we estimated 28 variables (20 demographic, 3 total scale scores, and 5 CBI subscales). We calculated the sample size as 7 times the number of variables and adjusted for a 20% potential invalid questionnaire rate. Thus, the final sample size was:


$$\begin{array}{l} 28\times 7\times 1.2=\;235.2 \end{array}$$

Rounded up, the sample size was 236.

### 2.5 Statistical analysis

Data processing was performed using SPSS 23.0 software. Descriptive statistics were expressed as frequencies and percentages for categorical variables and as the means ± standard deviations for normally distributed continuous variables or as medians and interquartile ranges for non-normally distributed continuous variables. Group comparisons were analyzed using the Mann–Whitney U-test and Kruskal–Wallis H-test. Correlational analysis was performed using Spearman's correlation analysis, while multiple linear regression analysis was performed for multivariate analysis. *p* < 0.05 was considered to indicate statistical significance.

## 3 Results

### 3.1 Demographic characteristics

The study included 236 IBD patients, ranging in age from 9 to 73 years, with a mean age of 32.11 ± 11.39 years. The cohort comprised 46 pediatric patients (under 18 years of age, 19.49%), 151 males (63.98%), and 85 females (36.02%). Regarding education, 72 patients (30.51%) held a bachelor's degree or higher. Employment status indicated that 107 patients were employed (45.34%), while 129 were not (54.66%). The patient population included 57 individuals with ulcerative colitis (24.15%) and 179 with Crohn's disease (75.85%). Disease duration was 5 years or longer in 62 cases (26.27%). In terms of disease activity, 142 patients (60.17%) were in remission, 27 (11.44%) were in a mild activity phase, 56 (23.73%) were in a moderate activity phase, and 11 (4.66%) were in a severe activity phase. Regarding insurance coverage, 138 patients (58.47%) had employee medical insurance, and 202 patients (85.59%) had purchased supplementary commercial insurance (Supplementary Table 1).

The caregivers (*n* = 236) ranged in age from 21 to 74 years, with a mean age of 45.39 ± 10.05 years. The caregiver group consisted of 78 males (33.05%) and 158 females (66.95%). The majority, 230 caregivers (97.46%), reported being in good physical health. In terms of education, 33 caregivers (13.98%) held a bachelor's degree or higher. Regarding employment status, 113 were employed full-time (47.88%), 41 were homemakers (17.37%), and 60 were retired (25.43%). Most caregivers, 234 (99.15%), reported having no religious beliefs, and 228 (96.61%) were married. In terms of their relationship with the patient, 128 caregivers (54.24%) were the patient's parents, and 91 (38.56%) were the patient's spouse. The monthly household income for 109 caregivers (46.19%) ranged from 5,001 to 10,000 yuan. A substantial proportion of caregivers, 172 (72.88%), had one co-caregiver and 203 caregivers (86.02%) provided care for < 8 h per day (Table 1).

**Table 1:** Basic demographic characteristics of IBD caregivers (*N* = 236).

**Variable**	**Number of cases**	**Percentage (%)**
**Age**		
< 30	19	8.05
30–39	38	16.10
40–49	98	41.53
50–59	66	27.97
>59	15	6.35
**Gender**		
Male	78	33.05
Female	158	66.95
**Health status**		
Unhealthy	6	2.54
Healthy	230	97.46
**Level of education**		
Junior high school and below	79	33.48
Above junior high school	124	52.54
Bachelor's degree and above	33	13.98
**Occupation**		
Students	3	1.27
Full-time employed	113	47.88
Part-time employed	19	8.05
Homemakers	41	17.37
Retired	60	25.43
**Religious affiliation**		
With religious beliefs	2	0.85
Without religious beliefs	234	99.15
**Marital status**		
Unmarried	5	2.12
Married	228	96.61
Widowed	3	1.27
**Relationship to patient**		
Spouse	91	38.56
Parents	128	54.24
Children	13	5.50
Siblings	2	0.85
Other relatives	2	0.85
**Monthly household income**		
< 5,000	36	15.25
5,001–10,000	109	46.19
>10,000	91	38.56
**Co-caregiver presence**		
None	45	19.07
1	172	72.88
≥2	19	8.05
**Daily care duration**		
< 8 h	203	86.02
8–12 h	28	11.86
12–24 h	5	2.12

### 3.2 SAS, SDS, PSQI, and CBI scores and incidence among IBD caregivers

**Figure 1:**
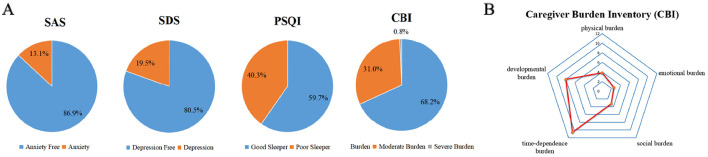
**(A)** Proportions of anxiety, depression, poor sleep, and severe burden in IBD caregivers. **(B)** Relative relationships of burden dimensions in the CBI scale.

The mean scores for IBD caregivers on the SAS, SDS, PSQI, and CBI were 36.16 ± 8.85, 41.51 ± 11.70, 5.48 ± 3.13, and 28.30 ± 13.91, respectively (Table 2). Radar chart analysis of the CBI subscales indicated that the highest-burden scores were related to time-dependent burden (10.56 ± 4.70) and developmental burden (8.02 ± 4.35; Table 2, Figure 1B). Based on SAS scores, 205 caregivers (86.90%) reported no anxiety, while 31 (13.10%) experienced anxiety. SDS scores revealed that 190 caregivers (80.50%) had no depression, whereas 46 (19.50%) experienced depression. PSQI scores indicated that 141 caregivers (59.70%) had good sleep quality, and 95 caregivers (40.30%) had poor sleep quality. Analysis of CBI scores showed that 161 caregivers (68.20%) experienced mild burden, 73 caregivers (31.00%) experienced moderate burden, and 2 caregivers (0.80%) experienced severe burden (Figure 1A).

**Table 2:** Scale scores of anxiety, depression, sleep, and caregiver burden in IBD caregivers (*N* = 236).

**Variable**	**Number of items**	**Score range**	**Mean scores**	**Mean item score**
SAS	20	25~100	36.16 ± 8.85	1.81 ± 0.44
SDS	20	25~100	41.51 ± 11.70	2.08 ± 0.59
PSQI	7	0~21	5.48 ± 3.13	0.78 ± 0.45
CBI	24	0~96	28.30 ± 13.91	1.18 ± 0.58
Physical burden	4	0~16	3.83 ± 3.70	0.96 ± 0.92
Emotional burden	5	0~20	2.00 ± 2.00	0.40 ± 0.40
Social burden	5	0~20	3.82 ± 3.26	0.76 ± 0.65
Time-dependence burden	5	0~20	10.56 ± 4.70	2.11 ± 0.94
Developmental burden	5	0~20	8.02 ± 4.35	1.60 ± 0.87

### 3.3 Correlation analysis between SAS, SDS, PSQI, and CBI scores among IBD caregivers

Spearman correlation analysis revealed a statistically significant positive correlation between the SAS, SDS, PSQI, and CBI scores among IBD caregivers (*r* = 0.180–0.709, *p* < 0.01; Table 3).

**Table 3:** Correlation analysis between SAS, SDS, PSQI, and CBI in caregivers of IBD patients (*N* = 236, *r*).

**Variable**	**CBI**	**Physical burden**	**Emotional burden**	**Social burden**	**Time-dependence burden**	**Developmental burden**
SAS	0.686^**^	0.693^**^	0.335^**^	0.584^**^	0.481^**^	0.489^**^
SDS	0.672^**^	0.709^**^	0.441^**^	0.555^**^	0.424^**^	0.482^**^
PSQI	0.591^**^	0.683^**^	0.180^**^	0.412^**^	0.485^**^	0.389^**^

^**^p < 0.01.

### 3.4 Comparison of CBI scores among caregivers with different characteristics

Significant statistical differences (*p* < 0.05) in caregiver burden scores were observed among IBD caregivers based on several factors, including the caregiver's age, gender, health status, education level, occupation, marital status, relationship with the patient, monthly household income, and daily caregiving duration. Similarly, significant differences (*p* < 0.05) in caregiver burden scores were found based on the patient's age, education level, occupation, disease type, disease duration, disease severity, method of medical expense payment, and the presence of supplementary commercial insurance (Table 4, Supplementary Table 2).

**Table 4:** Comparison of CBI scores by caregiver characteristics (*N* = 236).

**Variable**	**Median (IQR)**	**Z/H**	***P***
Age		13.513	0.009^**^
< 30	17 (14, 27.5)		
30–39	24.5 (15, 33)		
40–49	30 (21, 40)		
50–59	25.5 (18, 33)		
>59	22 (13, 29.5)		
Gender		−3.044	0.002^**^
Male	22 (16, 29)		
Female	28.5 (20, 40)		
Health status		−2.203	0.028^*^
Unhealthy	39.5 (28, 53)		
Healthy	25.5 (18, 38)		
Level of education		16.797	0.000^**^
Junior high school and below	29 (20, 41)		
Above junior high school	25 (19.5, 39)		
Bachelor's degree and above	16 (13, 27)		
Occupation		21.273	0.000^**^
Students	14 (7.50, 18.50)		
Full-time employed	25 (17, 33)		
Part-time employed	41 (15, 49.5)		
Homemakers	34 (28, 47.0)		
Retired	24 (18, 29.5)		
Religious affiliation		−0.088	0.93
With religious beliefs	29.5 (17, 42)		
Without religious beliefs	26 (18, 38)		
Marital status		6.188	0.045^*^
Unmarried	15 (14, 17)		
Married	26 (18, 38.5)		
Widowed	33 (27, 40.5)		
Relationship to patient		22.901	0.000^**^
Spouse	22 (15.5, 31.5)		
Parents	29 (22, 40.5)		
Children	19 (14, 28)		
Siblings	16.5 (10, 23)		
Other relatives	14.5 (12, 17)		
Monthly household income		25.619	0.000^**^
< 5,000	34 (20, 48.5)		
5,001–10,000	29 (22, 43)		
>10,000	21 (15, 28.5)		
Co-caregiver presence		3.187	0.203
None	23 (16, 32)		
1	27 (19, 39)		
≥2	23 (12.5, 40.5)		
Daily care duration		25.889	0.000^**^
< 8 h	24 (17, 33)		
8–12 h	40 (28.5, 48.5)		
12–24 h	50 (40, 53)		

^*^p < 0.05 (significant); ^**^p < 0.01 (highly significant).

### 3.5 Multivariate analysis of caregiver burden among IBD caregivers

In a multiple linear regression analysis, with all previously mentioned variables considered as independent variables and the CBI score as the dependent variable, the model (*R*^2^ = 0.645) revealed several significant associations. Higher levels of caregiver depression (*p* = 0.001) and anxiety (*p* = 0.001), as well as greater IBD disease severity in the patient (*p* = 0.000), were associated with increased caregiver burden. Furthermore, lower monthly family income (*p* = 0.009), younger patient age (*p* = 0.000), and lower patient educational level (*p* = 0.001) were also significantly associated with a heavier caregiver burden. Female caregivers reported a significantly higher burden compared to male caregivers (*p* = 0.049; Table 5).

**Table 5:** Multiple linear regression analysis describing the influencing factors of caregiver burden.

**Effect**	**β**	**SE**	**β'**	***t***	***P***	**95% CI**
Constant	3.654	4.568	-	0.800	0.425	(−5.347, 12.654)
Caregiver depression	0.329	0.098	0.277	3.362	0.001	(0.136, 0.522)
Disease severity	3.134	0.634	0.220	4.945	0.000	(1.885, 4.383)
Caregiver anxiety	0.453	0.130	0.288	3.484	0.001	(0.197, 0.709)
Monthly household income	−2.245	0.851	−0.113	−2.638	0.009	(−3.922, −0.568)
Patient age	−0.145	0.038	−0.160	−3.800	0.000	(−0.220, −0.070)
Patient education	−2.508	0.770	−0.137	−3.258	0.001	(−4.026, −0.991)
Caregiver gender	2.364	1.196	0.080	1.977	0.049	(0.008, 4.720)

*R*^2^ = 0.645, adj.*R*^2^ = 0.634, *F* = 59.216, *p* < 0.05.

### 3.6 Hierarchical regression analysis of the impact of IBD disease severity on caregiver burden levels

To further examine the impact of IBD disease severity on caregiver burden, a hierarchical regression analysis was conducted, using the CBI score as the dependent variable. In the first step, background variables of patients and caregivers (e.g., age, education level, occupation) were entered into the model. In the second step, disease severity was added as a variable. The results indicated that the variables in the first step explained 65.6% of the variance in caregiver burden. The addition of disease severity in the second step accounted for an additional 1.6% of the variance. Even after controlling for other variables, disease severity remained significantly associated with caregiver burden (*p* = 0.002; Supplementary Table 3).

### 3.7 Regression analysis of the impact of IBD disease type on caregiver burden levels

To further investigate the impact of specific IBD types on caregiver burden, patients were stratified into UC and CD subgroups. Separate multivariate linear regression analyses were performed for caregivers of UC patients and caregivers of CD patients. In each analysis, caregiver burden scores were used as the dependent variable, and the aforementioned relevant variables were included as independent variables.

Multivariate linear regression analysis for caregivers of CD patients (*R*^2^ = 0.643) revealed that higher levels of caregiver anxiety (*p* = 0.000) and depression (*p* = 0.011), greater CD disease severity in the patient (*p* = 0.000), and patient unemployment (*p* = 0.000) were associated with a significant increase in caregiver burden. Lower monthly family income (*p* = 0.002) and younger caregiver age (*p* = 0.005) were also significantly associated with a greater caregiver burden (Supplementary Table 4).

Multivariate linear regression analysis for caregivers of UC patients (*R*^2^ = 0.751) revealed that higher levels of caregiver depression (*p* = 0.000) and younger patient age (*p* = 0.000) were significantly associated with increased caregiver burden. Additionally, poorer caregiver health status (*p* = 0.016) and female caregiver gender (*p* = 0.029) also showed significant associations with greater caregiver burden (Supplementary Table 5).

## 4 Discussion

Caregiver burden is a significant concern for those supporting individuals with chronic illnesses. Experiencing excessive burden and distress can have detrimental consequences, including increased rates of depression and anxiety. The chronic, relapsing nature of IBD makes caregivers of IBD patients particularly vulnerable to this burden. Therefore, assessing and addressing caregiver burden in IBD is of paramount importance. However, research on the prevalence and impact of caregiver burden in IBD remains limited (23). To address this gap, this study employed a large-sample, cross-sectional survey focused specifically on caregiver burden in IBD, systematically evaluating the multidimensional challenges experienced by these caregivers.

Consistent with established risk factors for caregiver burden in other chronic illnesses (24–26), our findings indicate that caregiver burden in IBD is associated with patient age and educational level, as well as IBD severity and household financial strain. Furthermore, caregiver burden was significantly related to caregiver gender and psychological wellbeing, specifically anxiety and depression. Moreover, we identified unique aspects of caregiver burden in IBD compared to other chronic diseases. These included a higher burden among caregivers of CD patients compared to UC patients and a greater burden experienced by caregivers of pediatric IBD patients.

This study revealed a positive correlation between anxiety (SAS scores), depression (SDS scores), and poor sleep quality (PSQI scores) with caregiver burden (CBI scores). Specifically, higher levels of anxiety and depression were associated with greater caregiver burden, aligning with previous research that demonstrates the significant impact of psychological wellbeing on caregiver burden. Moreover, caregivers reporting poor sleep quality exhibited higher levels of burden, potentially due to long-term psychological stress and the demands of caregiving responsibilities leading to sleep disturbances. Furthermore, female caregivers experienced a significantly higher burden than male caregivers, which may be attributed to traditional gender roles that often assign greater caregiving responsibilities to women (9). These findings underscore the importance of considering the sociodemographic characteristics of both patients and caregivers when developing targeted support strategies to effectively alleviate caregiver burden.

Disease severity is a critical determinant of caregiver burden in IBD. In this study, we utilized the CDAI and MMS as established tools to assess disease severity. These scoring systems are widely employed in clinical practice to effectively reflect changes in disease status and treatment efficacy. Consistent with recent research, our results indicate that disease severity significantly impacts caregiver burden, even after controlling for other variables. Compared to caregivers of patients in remission, those caring for patients in the active phase of the disease experienced a significantly greater burden. This may be attributed to the fact that active disease necessitates more frequent and intensive caregiving, as well as increased psychological support and reassurance for the patient. These findings emphasize the importance of considering disease severity in IBD management and suggest that healthcare teams should provide targeted support to caregivers to mitigate their burden.

The specific type of IBD also influences caregiver burden, with caregivers of CD patients reporting a higher burden than those caring for UC patients. Although CD and UC are both classified as IBD, their distinct pathological features and clinical presentations may lead to varying degrees of caregiver burden. Caregivers of CD patients may experience greater psychological and financial strain due to the complexity of the disease, including its potential to affect a broader range of sites within the gastrointestinal tract and the higher incidence of complications such as perianal fistulas and intestinal strictures (27). Additionally, other factors, such as caring for pediatric patients and having caregivers with lower educational attainment, were associated with greater caregiver burden. Pediatric IBD patients often require more comprehensive medical care and psychological support, demanding a greater time and emotional investment from caregivers, which can intensify their burden. These findings suggest that younger patients and those with lower educational levels may require more intensive caregiving and supervision, potentially increasing the demands on their caregivers.

Household monthly income and healthcare payment methods also significantly impacted caregiver burden. Caregivers from lower-income households and those with lower insurance reimbursement rates experienced a greater burden, likely due to increased financial strain and limited access to essential healthcare resources (28, 29). Household income directly affects caregivers' ability to access and afford adequate social support networks, which is closely associated with their perceived burden (30). These findings underscore the importance of improving healthcare coverage and providing financial assistance to alleviate the economic burden faced by caregivers.

Caregivers of IBD patients face substantial psychological, financial, and social burdens. Reducing this burden not only enhances the caregivers' wellbeing but also improves patient outcomes. Therefore, caregiver support should extend beyond traditional medical interventions to encompass multidimensional support systems, with a particular emphasis on structured caregiver support programs and psychological interventions (31, 32). The following intervention strategies may help reduce caregiver burden:

1. Professional training and education: providing disease-specific training for caregivers can enhance their understanding of IBD symptoms, signs of disease exacerbation, treatment options, and effective daily management strategies. To enhance caregiver competence, we recommend developing standardized IBD nursing videos (15–20 min) distributed via hospital platforms (e.g., WeChat), covering: medication administration (oral/topical/injectable), nutrition monitoring and diet management, emergency symptom identification, specialist demonstrations (biological agent injection techniques, enteral nutrition tube care, nutritional formula preparation). These video resources should be supplemented with quarterly interactive Q&A sessions to address caregivers' individual concerns. This comprehensive approach improves caregiver confidence and coping abilities (33, 34), particularly for long-term caregivers. Regular implementation ensures accessibility and knowledge updates about medical advancements.
2. Strengthening social support networks. Social support is essential for caregivers (35). Establishing robust social networks allows caregivers to receive both emotional and informational support (36). For example, at our center, many patients have joined the IBD volunteer team, where IBD specialists and nurses lead regular meetings for peer support and online awareness campaigns. As a next step, we plan to establish an IBD Caregiver Support Group to enable caregivers to share experiences and receive both emotional and practical support. In China, the China Crohn's and Colitis Foundation (CCCF) has established collaborations with over 100 hospitals to provide IBD specialist consultations and patient groups. Expanding these initiatives to specifically include caregivers will provide a broader community for exchanging experiences and mutual support (37).
3. Improving access to medical services. Caregivers often experience significant time and financial burdens, particularly when frequent hospital visits are required. To address this, we have implemented multiple patient-centered measures, including dedicated WeChat groups for chronic disease management (involving both patients and physicians), remote online bed reservations, a fast-track endoscopy service, and online prescription services. Telemedicine services play a crucial role in alleviating this burden by facilitating remote appointments, online medical guidance, and electronic health record access, thereby reducing the need for caregivers to transport patients to hospitals (38). Additionally, our center has established a “two-way referral” collaboration with community hospitals, enabling IBD patients to receive primary care at local facilities while still benefiting from remote consultations with our IBD specialists.
4. Psychological support and interventions. Caregivers' mental health is a critical factor affecting their overall quality of life. Studies indicate that long-term caregiving responsibilities often lead to anxiety and depression (39). We propose a multi-tiered psychological support system: first, standardized scales (e.g., SAS, SDS) should be routinely administered during patient follow-ups to screen caregivers for psychological distress. Second, a hospital-community-family support network should be established, including hospital-based psychological counseling clinics and community caregiver support groups. Third, structured cognitive behavioral therapy (CBT) (40) should be provided to those with positive screening results, alongside basic CBT skills training for specialist nurses. Fourth, respite care services should be implemented in collaboration with social work departments to temporarily relieve caregiving burdens through short-term care solutions. Finally, regular mental health education workshops should be conducted to enhance caregivers' stress management skills. This comprehensive intervention program can effectively improve caregivers' psychological wellbeing and enhance their long-term caregiving adaptability.
5. Structured caregiver support programs: to effectively reduce caregiver burden, the implementation of structured support programs is necessary (41). These programs should encompass regular health monitoring, psychological counseling, opportunities for social interaction, and targeted disease-related training. Hospitals can organize informative lectures, facilitated group discussions, and personalized one-on-one counseling sessions to systematically equip caregivers with practical coping strategies. A well-designed, comprehensive support program can significantly enhance caregivers' ability to manage stress, reduce psychological burdens, and ultimately improve patient outcomes (42).

Several limitations should be acknowledged. First, as a cross-sectional survey, it only assesses caregiver burden at a single point in time, which limits our ability to understand the dynamic process of how caregiver burden changes in relation to disease progression in IBD patients. Although we attempted to cite longitudinal evidence, the scarcity of studies specifically examining caregiver burden in IBD caregivers hindered such integration. Future research should incorporate repeated measurements to track how burden fluctuates during transitions in disease activity (remission → relapse → remission). Second, the study was conducted at a single tertiary hospital, which may limit the generalizability of findings to broader populations or diverse healthcare settings. Third, while we identified several factors associated with caregiver burden, further validation is needed to confirm the effectiveness of proposed intervention strategies across different populations and contexts. Future studies should prioritize longitudinal and multicenter designs to explore dynamic changes in caregiver burden and its interplay with disease activity across varied geographical and clinical environments. Such work will provide more robust, widely applicable clinical guidance for IBD patients and their caregivers. Notably, longitudinal cohort studies on caregiver burden and their expansion into multicenter research are actively underway in our current work.

In conclusion, caregivers of IBD patients experience a multidimensional burden that significantly affects not only their quality of life but also potentially impacts patient outcomes. Therefore, assessing and alleviating caregiver burden is of critical importance. In the present study, we identified multiple factors affecting caregiver burden, including caregiver psychological status, disease severity in the patient, economic conditions, and specific caregiver characteristics. Based on these findings, we propose a series of targeted intervention strategies aimed at reducing caregiver burden and improving caregiver wellbeing. Future research should further investigate the effectiveness of these interventions and validate their impact across broader and more diverse populations.

## Data Availability

The original contributions presented in the study are included in the article/Supplementary material, further inquiries can be directed to the corresponding authors.
