# Supplementary material for: Exploring the multidimensional impact of caregiver burden in patients with inflammatory bowel disease
Source: Front Public Health. 2025 May 29;13:1528778. doi: 10.3389/fpubh.2025.1528778 (PMC12158650; doi:10.3389/fpubh.2025.1528778)
Supplement: Supplementary file 1 [file Table_1.docx]

| **Supplementary Table 1.** Basic demographic characteristics of IBD patient characteristics (N=236) | | |
| --- | --- | --- |
| Variable | Number of Cases | Percentage (%) |
| Age |  |  |
| <18 | 46 | 19.49 |
| 18-59 | 175 | 74.15 |
| >59 | 15 | 6.36 |
| Gender |  |  |
| Male | 151 | 63.98 |
| Female | 85 | 36.02 |
| Level of Education |  |  |
| Junior High School and Below | 64 | 27.12 |
| Above Junior High School | 100 | 42.37 |
| Bachelor's Degree and Above | 72 | 30.51 |
| Occupation |  |  |
| Employed | 107 | 45.34 |
| Out of Work | 129 | 54.66 |
| Type of IBD |  |  |
| UC | 57 | 24.15 |
| CD | 179 | 75.85 |
| Disease Duration |  |  |
| Less than 6 Months | 43 | 18.22 |
| 6 Months to 1 Year | 28 | 11.86 |
| 1 to 3 Years | 71 | 30.09 |
| 3 to 5 Years | 32 | 13.56 |
| 5 Years and Over | 62 | 26.27 |
| Disease Severity |  |  |
| Remission | 142 | 60.17 |
| Mild Activity | 27 | 11.44 |
| Moderate Activity | 56 | 23.73 |
| Severe Activity | 11 | 4.66 |
| Medical Insurance |  |  |
| Resident Medical Insurance | 98 | 41.53 |
| Employee Medical Insurance | 138 | 58.47 |
| Commercial Health Insurance |  |  |
| No | 202 | 85.59 |
| Yes | 34 | 14.41 |

| **Supplementary Table 2.** Comparison of CBI Scores among different characteristics of IBD patients (N=236) | | | |
| --- | --- | --- | --- |
| Variable | Median (IQR) | Z/H | *P* |
| Age |  | 14.376 | 0.001** |
| <18 | 32.5(24, 47) |  |  |
| 18-59 | 24(17, 33) |  |  |
| >59 | 26(16, 31.5) |  |  |
| Gender |  | -1.794 | 0.073 |
| Male | 27(19, 41) |  |  |
| Female | 24(17, 31) |  |  |
| Level of Education |  | 9.657 | 0.008** |
| Junior High School and Below | 30.5(19, 43.5) |  |  |
| Above Junior High School | 27(18.5, 39.5) |  |  |
| Bachelor's Degree and Above | 22.5(16.5, 29) |  |  |
| Occupation |  | -4.059 | 0.000** |
| Employed | 22(16, 29) |  |  |
| Out of Work | 30(21, 42) |  |  |
| Type of IBD |  | -2.126 | 0.034* |
| UC | 23(16, 31) |  |  |
| CD | 27(19, 40) |  |  |
| Disease Duration |  | 30.199 | 0.000** |
| Less than 6 Months | 40(26, 49) |  |  |
| 6 Months to 1 Year | 31(21.5, 38.5) |  |  |
| 1 to 3 Years | 26(16.5, 32) |  |  |
| 3 to 5 Years | 25(20.5, 30.5) |  |  |
| 5 Years and Over | 19(15, 29) |  |  |
| Disease Severity |  | 8.393 | 0.015* |
| Remission | 22(16, 30) |  |  |
| Mild Activity | 23(21,28) |  |  |
| Moderate Activity | 39.5(28,47) |  |  |
| Severe Activity | 54(51,61) |  |  |
| Medical Insurance |  | -5.353 | 0.000** |
| Resident Medical Insurance | 32.5(23, 47) |  |  |
| Employee Medical Insurance | 22(16, 29) |  |  |
| Commercial Health Insurance |  | -3.204 | 0.001** |
| No | 27(19, 40) |  |  |
| Yes | 20.5(16, 26) |  |  |
| **p*<0.05(significant)*；**p*<0.01(highly significant) | | | |

| **Supplementary Table 3.** Hierarchical Regression Analysis of Disease Severity on Caregiver Burden | | | | |
| --- | --- | --- | --- | --- |
| Variable | Model 1 | | Model 2 | |
|  | β | P | β | P |
| Patient Age | -0.635 | 0.671 | -1.412 | 0.342 |
| Patient Education | -0.564 | 0.545 | -0.509 | 0.577 |
| Patient Occupation | 0.552 | 0.696 | 0.870 | 0.530 |
| Type of IBD | 1.565 | 0.284 | 1.498 | 0.295 |
| Disease Duration | -0.946 | 0.051 | -0.558 | 0.253 |
| Medical Insurance | -1.836 | 0.243 | -1.876 | 0.223 |
| Commercial Health Insurance | -0.938 | 0.584 | -0.621 | 0.712 |
| Caregiver Age | -0.242 | 0.787 | -0.259 | 0.768 |
| Caregiver Gender | 2.847 | 0.039 | 2.602 | 0.054 |
| Caregiver Health Status | -3.380 | 0.371 | -2.057 | 0.580 |
| Caregiver Education | -3.133 | 0.006 | -2.821 | 0.012 |
| Caregiver Occupation | -1.031 | 0.066 | -0.725 | 0.193 |
| Caregiver Marital Status | -4.292 | 0.070 | -3.462 | 0.137 |
| Relationship to Patient | -1.752 | 0.028 | -1.619 | 0.039 |
| Monthly Household Income | -1.928 | 0.047 | -1.810 | 0.057 |
| Daily Care Duration | 3.161 | 0.043 | 2.103 | 0.178 |
| SAS | 0.440 | 0.002 | 0.411 | 0.003 |
| SDS | 0.291 | 0.007 | 0.266 | 0.013 |
| PSQI | 0.612 | 0.038 | 0.517 | 0.073 |
| Disease Severity |  |  | 2.238 | 0.002 |
| F | 21.692 | ＜0.001 | 22.014 | ＜0.001 |
| R² | 0.656 |  | 0.672 |  |
| Adjusted R² | 0.626 |  | 0.641 |  |
| ΔR² | 0.656 |  | 0.016 |  |
| ΔF | 21.692 | ＜0.001 | 10.331 | 0.002 |

| **Supplementary Table 4.** Multivariate Linear Regression Analysis of Factors Influencing Caregiver Burden in CD Patients | | | | | | |
| --- | --- | --- | --- | --- | --- | --- |
| Effect | β | SE | β’ | t | P | 95%CI |
| Constant | 1.317 | 5.844 | - | 0.225 | 0.822 | (-10.218,12.853) |
| Caregiver Anxiety | 0.531 | 0.149 | 0.341 | 3.556 | 0.000 | (0.236,0.826) |
| Disease Severity | 2.822 | 0.729 | 0.198 | 3.870 | 0.000 | (1.382,4.261) |
| Patient Occupation | 5.113 | 1.349 | 0.178 | 3.789 | 0.000 | (2.45,7.777) |
| Monthly Household Income | -3.284 | 1.021 | -0.158 | -3.217 | 0.002 | (-5.299,-1.269) |
| Caregiver Age | -0.201 | 0.070 | -0.135 | -2.874 | 0.005 | (-0.34,-0.063) |
| Caregiver Depression | 0.292 | 0.114 | 0.244 | 2.561 | 0.011 | (0.067,-0.517) |
| *R*2=0.643，adj.R2=0.630,F=51.535,p＜0.05 | | | | | | |

| **Supplementary Table 5.** Multivariate Linear Regression Analysis of Factors Influencing Caregiver Burden in UC Patients | | | | | | |
| --- | --- | --- | --- | --- | --- | --- |
| Effect | β | SE | β’ | t | P | 95%CI |
| Constant | 17.134 | 10.619 | - | 1.614 | 0.113 | (-4.175,38.443) |
| Caregiver Depression | 0.822 | 0.080 | 0.734 | 10.257 | 0.000 | (0.661,0.982) |
| Patient Age | -0.201 | 0.053 | -0.279 | -3.819 | 0.000 | (-0.306,-0.095) |
| Caregiver Health Status | -11.399 | 4.581 | -0.178 | -2.488 | 0.016 | (-20.591,-2.206) |
| Caregiver Gender | 3.840 | 1.706 | 0.157 | 2.251 | 0.029 | (0.416,7.263) |
| *R*2=0.751*，adj.R*2=0.732*,F*=39.223*,p*＜0.05 | | | | | | |
